# Vibration Behavior of 3D-Printed Graded Composites: Fabrication and Testing

**DOI:** 10.3390/polym16233428

**Published:** 2024-12-06

**Authors:** Fazeel Khan, Kumar Singh, Justin Carter

**Affiliations:** Department of Mechanical and Manufacturing Engineering, Miami University, Oxford, OH 45056, USA; singhkv@miamioh.edu (K.S.); carterjb@miamioh.edu (J.C.)

**Keywords:** 3D printing, graded materials, vibration analysis

## Abstract

Multi-head 3D printers afford the ability to create composite structures with significant differences in properties compared to those created through traditional molding techniques. In addition to the usage of different viscoelastic polymeric materials, the selective spatial placement of the build materials enables the creation of layered and graded geometries to achieve specific mechanical and/or vibrational characteristics. This paper describes how the mechanical properties of the individual materials can be used to predict the damping and natural frequencies of a 3D-printed graded structure. Such structures can find usage in rotating machinery, beams, etc., where vibrational characteristics must be controlled. The simulation and experimental results are presented and two forms of the storage and loss modulus are considered: fixed and variable. For the latter condition, *E*′ and *E*″ are established as functions of temperature and frequency. Modal vibration testing of the graded samples shows a good match between the simulation and experimental trials, thereby supporting the proposed model as a useful tool for prescribing the structure of a printed part with tailored dynamic properties.

## 1. Introduction

Fused deposition modeling (FDM) is a type of 3D printing technology that allows for the rapid prototyping of complex structures, and it has impacted engineering in many industries [1,2,3]. This low-cost solution allows for the production of prototypes with complex geometries, internal features, etc., because traditional assemblies consisting of several components and/or sub-assemblies can often be printed as a single part. Cost saving is achieved by avoiding the need for high-cost casting molds or forging dies if traditional manufacturing practices are used. The design of functional structures can require the implementation of multiple materials to achieve the desired performance. The integration of materials with differing properties can help fulfill various performance requirements such as stiffness, load-carrying capacity, deflection, and vibrational behavior. The integration of multiple materials allows composite structures to have improved functionality over one made of a single material and allows for the research and design of structures not previously explored. In this research, 3D-printed axially graded plates will be fabricated from multiple materials using the FDM process such that their vibrational performance can be evaluated.

To predict the vibration characteristics of structural components (beams, plates, etc.), the inherent nonlinear temperature and frequency dependency of material properties present in viscoelastic polymeric materials, commonly used for 3D printing, needs to be investigated. The FDM process typically uses polymers that are often modeled as viscoelastic materials that exhibit inherent elastic and damping behaviors [4,5]. These materials require characterization based upon various parameters such as temperature, strain, strain rate, and frequency. In this research, systematic testing was performed to characterize the static and dynamic behaviors of polymeric materials. Subsequently, material models were used for the computational modeling of various graded structures through finite element (FE) simulations. Finally, the experimental testing of fabricated graded structures via FDM 3D printing was performed to validate the response obtained from computational modeling. This research contributes to the understanding of the structural performance of 3D-printed graded polymeric structures and associated computational modeling vis-à-vis the design of functional parts.

Within the literature, a common form of multiple-material structures consists of discrete layered composites. These materials have been well studied for their mechanical properties and behaviors in many applications. Specifically, the vibration of thin and thick laminated composite rectangular plates has been explored [6]. Similar to the scope of this research project, the vibration of thin laminated structures composed of viscoelastic smart materials has also been explored [7].

Functionally graded materials (FGMs) are also common multiple-material structures studied in the literature. FGMs consist of a smooth distribution between different materials rather than discrete layers. For plate structures, the material distribution most commonly varies through thickness. However, the recent literature has expanded to material distributions along the major axes (the length and width of a plate). Specifically, a finite element (FE) method approach for FGM plates subjected to transverse loading with thickness and lengthwise material distribution has been proposed in the literature [8]. The modal analysis of FGM plates with material distributions along the thickness has been explored similarly using an FE approach [9]. Furthermore, modal vibration optimization (maximizing the fundamental frequency) has been explored for such FGM structures with multiple-material distributions along the major axes [10]. Note that this study pertains primarily to linear elastic materials such as aluminum and steel.

As previously mentioned, the literature pertaining to multiple-material 3D-printed structures is present but limited. The properties of discretely graded (layered) 3D-printed parts manufactured using a Polyjet process are explored in [11]. The Polyjet process consists of micrometer-sized polymer droplets that are cured layer by layer by UV light. This article explored graded material patterns along the thickness and width of small rectangular beam and plate samples. The purpose of this article was to show how the volume fraction, temperature, and print orientation could influence the mechanical properties of these multiple-material samples [11]. Another article of relevance explored the buckling behavior of FDM 3D-printed FGM beams. These beams were constructed with common FDM polymers such as polylactic acid (PLA), nylon, and acrylonitrile butadiene styrene (ABS). This article explored the influences of multiple grading functions along the major axes of the beam on its buckling behaviors [12]. The literature related to the experimental investigation of the vibration performance of spatially graded (along its major axes) viscoelastic polymeric structures is limited and available for axially and transversely vibrating beam-type structures [13,14]. Therefore, in this research, the vibration characteristics (natural frequencies and damping) of thin spatially graded FDM 3D-printed plates with discrete distributions of viscoelastic polymeric materials were investigated. Numerical simulations using an in-house developed FE model for thin graded viscoelastic plates were used to investigate the vibration behavior of these structures with different grading schemes and subject to different temperatures.

Single-material FDM 3D-printed structures have been explored in the literature regarding mechanical properties and applications. However, 3D-printed graded structures with multiple materials are not well studied, especially for vibration characteristics. As previously mentioned, the literature related to the experimental investigation of the vibration performance of 3D-printed spatially graded (along its major axes) viscoelastic polymeric structures is limited and available for axially and transversely vibrating beam-type structures [13,14]. Thus, this research also aims to propose a modeling approach for the vibration of 3D-printed viscoelastic plates with a nonlinear frequency dependency on material properties and spatial grading patterns along the major axes.

## 2. Materials and Methods

### 2.1. Preparation of 3D-Printed Polymer Samples

**Figure 1:**
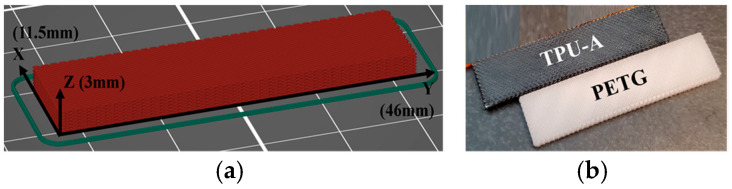
(**a**) DMA specimen in slicing software with labeled axes and dimensions, and (**b**) physical 3D-printed DMA samples.

In order to characterize dynamic material properties using DMA, 3D-printed samples of the materials were produced. These samples were fabricated with fused deposition modeling using the Prusa i3 MK3s (Prusa Research, Partyzánska, Czech Republic) equipped with an MMU2s auxiliary. This unit is capable of producing both single- and multiple-material parts with a single nozzle using the filament switching capability of the MMU2s attachment. For the purposes of constructing axially graded composites, two materials were selected based upon criteria including toughness, flexibility, adhesion to each other, and printable temperature ranges: (a) Polymaker PolyLite (Polymaker, Missouri City, TX, USA) polyethylene terephthalate glycol (PETG) and (b) NinjaTek Cheetah (Fenner Precision Polymers, PA, USA) thermoplastic polyurethane (TPU-C) with 95A hardness. The DMA specimen dimensions were chosen to be 11.5 × 46 × 3 mm as shown in the screenshot of the specimen in the printer slicing software (PrusaSlicer 2.6) with the respective dimensions and axes, which can be seen in Figure 1.

**Figure 2:**
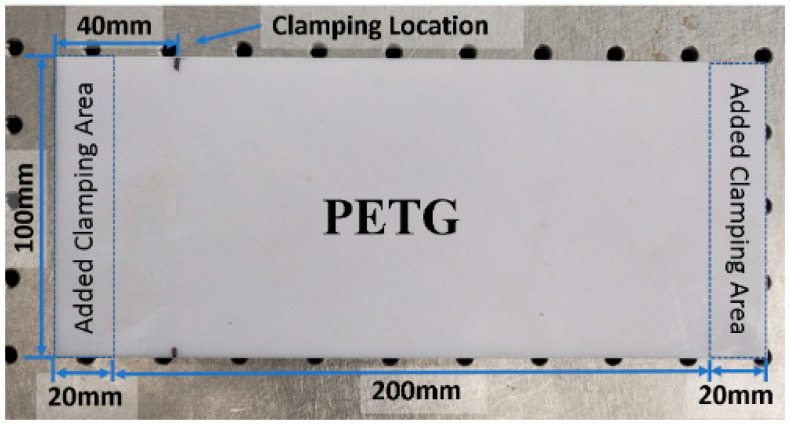
Plate specimen made of PETG with overlaid dimensions.

In order to fabricate a graded plate for subsequent vibration analysis in cantilever configurations, a CreatBot F430 (Henan CreatBot Technology Ltd., Zhengzhou, China) printer was used to fabricate the plate sections of both PETG and TPU-A materials. The following nominal dimensions were selected: 100 × 200 × 4 mm. It was decided to add an additional 20 mm onto both 100 mm sides for clamping purposes, thereby making the total length 240 mm. The dimensions and layout of a typical sample are shown in Figure 2.

**Figure 3:**
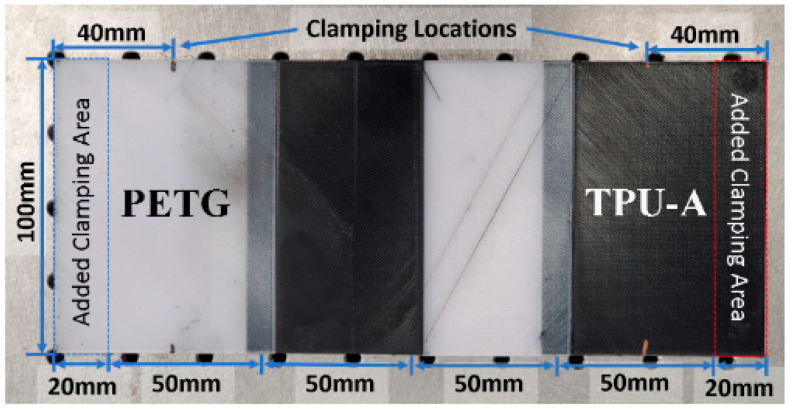
Full-scale graded plate with two sections of PETG and TPU-A.

The axially graded plates were then fabricated by combining equal-length plate sections of both materials along the 200 mm side, as shown in Figure 3 with overlaid dimension labels.

**Figure 4:**
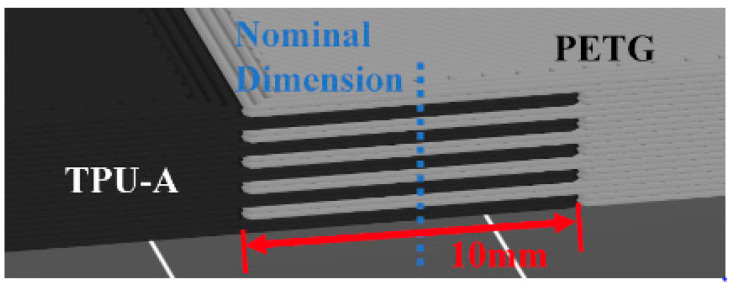
Tongue and groove joint between plates (layered PETG/TPU-A).

The individual plates were joined using a 10 mm tongue and groove arrangement with a Loctite plastic epoxy applied to the area to prevent separation at the joint (see Figure 4). A DMA specimen for this layered PETG/TPU-A section was also created to perform a subsequent dynamic characterization of material properties to be used for numerical simulation and experimental validation studies.

### 2.2. Dynamic Material Characterization of DMA Samples

**Figure 5:**
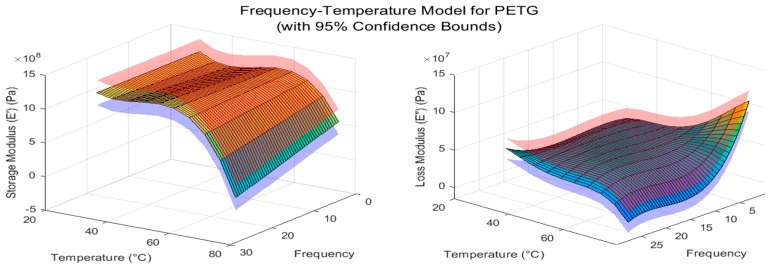
Temperature and frequency curve-fitted plots with confidence bounds for estimating *E*′(*ω*,*T*) and *E*″(*ω*,*T*) for PETG. The color surface contour plots mark the 95% confidence interval.

A TA Instruments dynamic mechanical analyzer (DMA model RSA-III, TA Instruments, New Castle, DE, USA) was used to perform a dynamic analysis on the materials. The 40 mm length three-point-bending fixture was selected for the analysis. Temperature and frequency sweeps from 1 to 30 Hz and 30 to 75 °C were performed to estimate the frequency- and temperature-dependent storage modulus *E*′ and loss modulus *E*″ of the materials. Three DMA specimen samples of PETG, TPU-A and layered PETG/TPU-A were used for dynamic characterization. In order to obtain the continuous functions of *E*′(*ω*,*T*) and *E*″(*ω*,*T*), the curve fitting tool in MATLAB^®^ (version R2022b) was used for the frequency–temperature sweep data, and the representative fitted curve for the PETG sample is shown in Figure 5.

The curve fitting allows the extraction of material parameters as a function of fixed frequency and variable frequency models at any given temperature of interest. For example, for the room temperature values (23 °C), the constant material properties for the quasi-static loading (@ 12.5 Hz) are shown in Table 1.

Similarly, a frequency-dependent material model can be obtained for a given temperature. For example, a frequency-dependent linear material model in the form

(1) $$\begin{array}{l} E^{\prime }\left(\omega \right)=p_{1}\omega +p_{2}\\ E^{\prime \prime }\left(\omega \right)=p_{3}\omega +p_{4} \end{array}$$

can be obtained. The parameters of the linear material model (1) for room temperature is provided in Table 2.

### 2.3. Experimental Estimation of Poisson’s Ratio

Poisson’s ratio for the polymers of interest can vary depending on the printing variables. Thus, it was necessary to determine this value through the experimental testing of 3D-printed specimens [15]. Poisson’s ratio for most polymeric materials varies between 0.3 and 0.5 [16], so an arbitrary Poisson’s ratio of 0.3 has been used as a starting value for preliminary modeling [15]. From the experimental testing data, the Poisson’s ratio for a material can be found by calculating the ratio of transverse to axial strain in the sample (see Equation (2)).

(2) $$v=-\frac{\varepsilon_{transverse}}{\varepsilon_{axial}}$$

**Figure 6:**
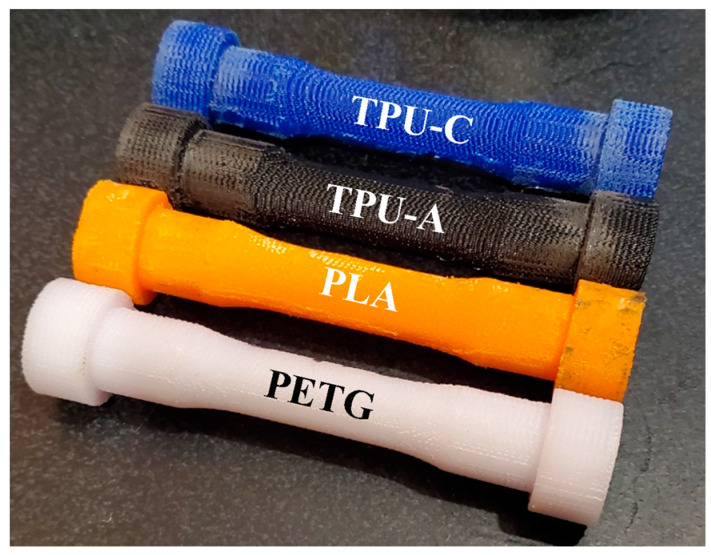
Poisson’s ratio testing samples.

In this study, for greater accuracy, the experimental testing of each 3D-printed sample was performed on an MTS biaxial servo-hydraulic test frame equipped with both axial and transverse extensometers. The samples were designed to be used with custom split collars designed for soft polymer materials. The samples are shown in Figure 6.

The samples were tested to an axial strain of 2%, and the Poisson’s ratio of the material was obtained from the sample behavior from 0% to 1% of this test. This property can change for high material strain, which is outside the scope of this characterization. The resulting Poisson’s ratios found for each of the tested materials are provided in Table 3.

These material properties are subsequently used for the numerical simulation for axially graded beams in computing the natural frequencies and damping ratios associated with the vibration modes for estimating the dynamic performance of the structure and in validating the experimental observations.

### 2.4. Mathematical Modeling for Vibration Performance of Graded Plates

In order to evaluate the vibration performance of graded plates, an in-house finite element code was developed. A finite element (FE) model was developed based on the Mindlin plate theory and the formulation of free vibration characteristics of the graded plates led to a system of equations in the following form,

(3) $$M\ddot{w}+K\left(\left(\omega ,T\right)\right)w=0$$

where M is the global mass matrix, $K\left(\left(\omega ,T\right)\right)$ represents the global stiffness accommodating frequency- and temperature-dependent material properties, such as complex elastic modulus, $E*(\omega ,T)=E^{\prime }(\omega ,T)+iE^{\prime \prime }(\omega ,T)$, and w is the degrees of freedom defining the vibration co-ordinates of the graded plate. The details of this FE model are presented in [17,18], which have been verified for the model’s accuracy for known analytical solutions of plates. The stiffness matrices associated with the Mindlin plate model account for shear effects and are functions of Poisson’s ratio. Details of the mesh size, its convergence, and the accuracy of the in-house finite element code are provided in [17,18]. While comparing the accuracy of the FE model with analytical solutions for a plate in [18], it is shown that it provides acceptable errors in eigenvalue computation (0.34% error for the fundamental frequency for 975 DoF and 0.01% error for 2583 DoF). Therefore, for computational efficiency in solving associated linear (the fixed modulus case) and nonlinear eigenvalue problems (for the frequency-dependent modulus), different element sizes and hence DoF values are chosen for the analysis and comparison purposes in the subsequent sections while ensuring an acceptable range of prediction errors.

System (3) leads to the following characteristic nonlinear complex eigenvalue,

(4) $$\;\begin{array}{cc} \left(K_{e}^{*}(\lambda ,T)-\lambda M_{e}\right)u=0\;\to & \;A\left(\lambda \right)u=0 \end{array}$$

The complex eigenvalues $\lambda_{i}$, for *i* = 1, 2, … n, are a function of natural frequencies $\omega_{i}$ and damping ratio $\zeta_{i}$ associated with an individual *n* number of modes of vibration. In our analysis, for the constant room temperature the transcendental eigenvalue problem (4) has frequency-dependent system matrix $A\left(\lambda \right)$.

For the purpose of analyzing the dynamic behavior of viscoelastic structures, various modeling techniques are used. The dynamic characteristics are typically obtained by fitting the experimental response in a time or frequency domain in computing complex moduli of the materials. Different models take varying approaches in how these material parameters are integrated for dynamic or vibration analysis. For example, rational polynomial models, such as Golla–Hughes–McTavish’s [19], have been developed, which add degrees of freedom to represent internal energy dissipation mechanisms. Similarly, anelastic displacement field models [20] capture frequency-dependent damping characteristics, and fractional derivative models [21] are capable of capturing both frequency- and time-dependent characteristics. Different models balance the accuracy and complexity of solution strategies while facilitating different ways to incorporate them in finite element or state-space models for the subsequent dynamics and control analysis, as summarized in this extensive review paper [22]. In this research, the complex modulus of the materials is directly integrated into the structural analysis, leading to a frequency-dependent nonlinear eigenvalue problem (4) for which the authors have developed numerical solutions strategies in [23,24]. The Newton’s eigenvalue iteration method developed in [24] is used here to compute the eigenvalues and associated natural frequencies $\omega_{i}$ and damping ratio $\zeta_{i}$, which are shown to be computationally effective and accurate.

### 2.5. Vibration Testing of Graded Structures

**Figure 7:**
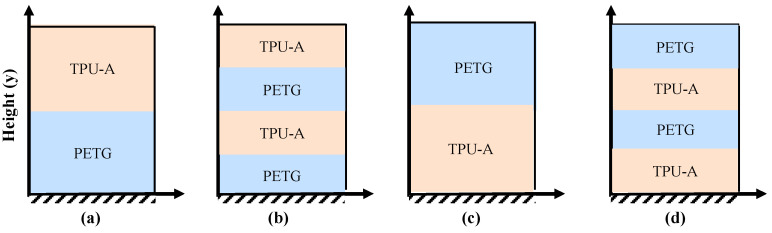
Grading scheme for experimental specimen: (**a**) PETG/TPU-A 1y, (**b**) PETG/TPU-A 2y, (**c**) TPU-A/PETG 1y, and (**d**) TPU-A/PETG 2y.

The experimental vibration testing of a 3D-printed axially graded structure was conducted to evaluate the dynamic performance and to validate the mathematical modeling in computing natural frequencies and damping ratios of graded beams. The full-scale single-material and graded plates were fabricated for modal vibration testing. For simplicity, the plates were designed to be tested with a cantilever boundary condition. This allows the evaluation of a total of four grading schemes associated with graded plates, as seen in Figure 3. For the graded beam, vibration testing was performed with the long axis of the specimen in the vertical (*y*) direction. The following labeling convention identifies the configuration of clamped materials and number of materials defining the grading and is subsequently used for the analysis, testing and comparison: a specimen designated PETG/TPU-A 1y refers to the graded plate with one section of each material and 40 mm of the PETG section clamped into the vise, as illustrated in Figure 7.

**Figure 8:**
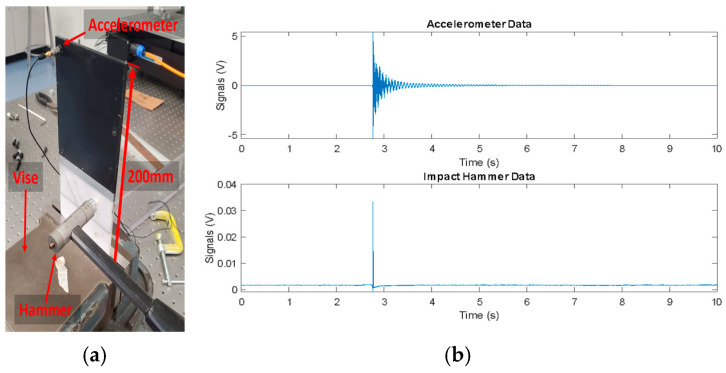
(**a**) Vibration testing setup and (**b**) representative input and output data from vibration testing.

Finally, the plate specimens were clamped into a bench vise such that the nominal dimension (100 × 200 × 4 mm) was cantilevered upwards with 40 mm of the specimen clamped in the vise. A PCB 086C03 (PCB Piezotronics, Depew, NY, USA) impact hammer and a PCB U352C66 ICP (PCB Piezotronics, USA) accelerometer were used for the vibration testing of the plates. The accelerometer was mounted to the upper corner of the specimen, and the specimen was excited with the impact hammer. For example, the testing setup for grading scheme TPU-A/PETG 1y shown in Figure 7c can be seen in Figure 8a.

**Figure 9:**
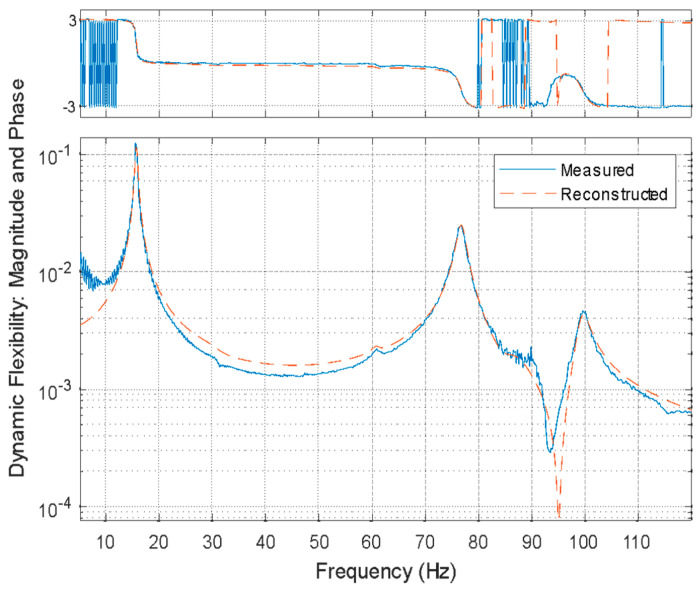
A representative curved fitted frequency–response function from vibration testing.

For each plate orientation associated with different grading schemes in Figure 7, four modal vibration tests were performed where the plate was excited with the impact hammer force and the accelerometer was used to obtain the response of impact testing. A representative input (hammer excitation) and representative response (acceleration data) are shown in Figure 8b. For each test, frequency–response function (FRF) values were extracted and curve fitted using the MATLAB^®^ vibration toolbox. From the curve-fitted data, the natural frequencies associated with any given modes of vibration and associated damping ratios were extracted. For example, a representative curve-fitted FRF shown in Figure 9 allows the estimation of the natural frequencies and damping ratios of the first three modes of vibration of the graded plate.

## 3. Results

### Vibration Performance of Polymeric Plates

The natural frequencies and damping ratios associated with cantilever plates made of single materials (PETG or TPU-A) were computed by solving Equation (4) with fixed and frequency-dependent material parameters listed in Table 1, Table 2 and Table 3. For simulation purposes, the mean values and upper and lower bounds of material properties were selected to capture the influence on vibration performance due to the variations in material parameters. These values, associated with the first three modes of vibration, are compared in Table 4 with those obtained from vibration modal testing. The variation in the experimental results shows the mean values and corresponding standard deviations of four vibration test data for each plate testing.

Similarly, by modifying the in-house FE code, the natural frequencies and damping ratios associated with different configurations of graded plates, as shown in Figure 7, are computed and compared with the experimental results in Table 5.

The difference between the predicted natural frequencies from the model and experimental natural frequencies shows that they vary based on the material, the grading scheme, boundary condition, and the selection of material model type. For example, experimental natural frequencies for the plates made of single materials are generally matched more closely by the frequency-dependent modulus model compared to the fixed modulus model, particularly in the first and second modes. The frequency-dependent material models perform slightly better overall in predicting the first two natural frequencies of the structures. Graded plates exhibit natural frequencies that generally fall between the bounds of single-material plates, with PETG/TPU-A grading schemes showing slight shifts depending on the layer configuration (1y or 2y). Frequency-dependent models perform better across all graded schemes, especially in the first two modes. The errors in the third mode do not show this distinction, which has a higher error rate for both material models. This is likely because the DMA material samples were only tested up to 30 Hz for dynamic characterization, and the frequency-dependent material model does not account for high frequencies.

It is observed that the experimental natural frequencies and the associated predictions from frequency-dependent models have higher values compared to the fixed modulus case. This can be contributed to the storage modulus being higher as the frequency increases in the selected material model (1) as stiffness increases linearly with frequency. Similar increases in natural frequencies are also observed in the analysis of composite sandwich plates composed of viscoelastic materials [25]. It is important to note that the observed material properties are consistent with the observations made in [26] on 3D-printed beams, in which an increase in the natural frequency was also seen to accompany an increase in stiffness. It is also seen that experimental damping ratios as well as frequency-dependent damping ratios decrease compared to the case of the fixed modulus due to a decrease in the ratio between the storage and the loss modulus for the selected material model (1). This can be contributed to lower energy dissipation, especially at higher frequencies. Models incorporating a fixed modulus rather than a frequency-dependent form cannot capture these trends in natural frequencies and damping ratios, especially in predicting high-mode vibrations.

## 4. Discussion

The vibration analysis and performance of viscoelastic structures have been studied widely by utilizing various modeling techniques, which includes a vibration analysis of viscoelastic sandwich beams [27] for constant and frequency-dependent material properties and various boundary conditions and a finite element analysis of composite beams [28] and the vibration of viscoelastic plates [29]. Typically, the treatment of viscoelastic materials configured as the center layer in sandwich beams [30,31] and their usage as a constrained layer [32] is far more common. The analysis developed here is unique regarding the way in which the material is distributed along the axis of the beam rather than in the direction of the thickness. This allows for the potential of a more optimal distribution of viscoelastic materials in structures to achieve the desired performance.

In this study, we observe the importance of incorporating frequency dependence on modulus properties. It is shown that they capture the true trends of increase in natural frequencies and decrease in damping ratios, especially for higher modes of vibrations. It also highlights variations in natural frequencies and damping ratios for different axially grading schemes, which could allow the selection of one grading scheme over another based on the desired frequency response and dissipation strategy. Damping ratios for single-material plates show significant differences between two materials. TPU-A exhibits consistently higher damping, with experimental values around 6% across modes, while PETG is closer to 1–2%. For all cases, the damping ratios are small, ranging from 1 to 7%, having large variations from their mean values. Frequency-dependent models also accurately captured damping trends for the first two modes, unlike the fixed-modulus model, which predicted a constant damping ratio across all modes, as expected. Damping trends for graded plates are unique to each scheme, with PETG/TPU-A graded plates exhibiting damping ratios that generally fall within the bounds of single-material plates. The frequency-dependent model more accurately reflects observed mode-specific damping behaviors, providing distinct damping values across modes rather than a constant damping ratio. Overall, the FE models effectively predicted natural frequencies and captured modal damping ratio trends for 3D-printed single-material and axially graded plates.

Potential sources of discrepancies in the results include fabrication and testing variations, for example, variations in printing parameters when printing the DMA samples for the material characterization and fabrication of larger plate segments. Experimental setup inconsistencies, particularly with ‘ideal’ clamping conditions for vibration testing, may also have introduced variability. Overall, frequency-dependent models demonstrate greater accuracy in predicting both natural frequencies and damping trends, especially when material distributions vary, highlighting their utility in modeling polymeric materials and specially graded material structures. Incorporating frequency-dependent moduli is critical for improved predictions of mode-specific damping and natural frequencies as they can influence the dynamic performance and stability of viscoelastic systems [33] and could benefit in engineering applications involving broad frequency excitations.

## 5. Conclusions

The vibrational characteristics of 3D-printed graded plates made of multiple polymers were analyzed by developing an FE analysis tool for viscoelastic plates. The viscoelastic material properties of 3D-printed polymers were obtained using DMA testing. These properties were used in the FE tool for computing the vibration spectral characteristics (natural frequency, damping ratio, and mode shapes) of graded plates. Six different grading schemes (three each in the length and width directions) using two polymeric materials were evaluated for their vibration performance. It was shown that grading patterns with multiple materials allowed for the natural frequencies and damping characteristics of a structure to be altered. The graded pattern of the structure also influenced the mode shapes by changing the modal displacement intensity throughout the plate. The bounds for the vibrational characteristics of multiple-material structures were shown to be that of single-material plates made of each polymer. Temperature-dependent polymer properties were explored, and the unique softening and damping characteristics of each material were shown to influence the natural frequencies, damping ratios, and mode shapes of plates. Finally, the frequency-dependent properties of the viscoelastic materials were shown to further influence the vibrational performance of both single-material and graded plates.

Full-scale single- and multiple-material graded plates were fabricated and tested for their spectral characteristics. It was found that the proposed modeling techniques were able to model vibration characteristics such as the natural frequencies and trends in the damping ratios of the 3D-printed structures. It was also found that the modeling of frequency-dependent material properties provided additional modeling accuracy and insights into the behavior of 3D-printed structures, such as the damping ratios of single-material plates. However, modeling limitations were encountered, which include variations in material fabrication and the testing setup, as well as limitations from extrapolating the frequency-dependent models to higher frequencies. Sources of the variation were reflected upon, and methodologies were proposed for improving the modeling results for future investigations.

## Figures and Tables

**Table 1:** Fixed modulus material properties for full-scale plate predictions.

Material	E′ (MPa) {95% CI Lower, Upper}	E″ (MPa) (95% CI Lower, Upper)
PETG	1112.3 {1099.2, 1126.6}	37.48 {32.58, 42.37}
TPU-A	712.2 {569.2, 855.2}	110.9 {93.08, 128.8}
Layered PETG/TPU-A *	923.2 {846.3, 1000.4}	81.48 {70.56, 92.41}

* Assuming the average value for the following quantities: ρ = 1235 kg/m^3^, υ = 0.45.

**Table 2:** Frequency-dependent material parameters for full-scale plate predictions. The results are presented as follows: average {95% CI lower, upper}.

Material	$p_{1}$ (kPa)	$p_{2}$ (MPa)	$p_{3}$ (kPa)	$p_{4}$ (MPa)
PETG	340.3 {291.8, 388.8}	1086 {1082, 1091}	21.42 {4.069, 38.77}	35.79 {34.12, 37.46}
TPU-A	1335 {973.1, 1697}	607.3 {574.5, 640.2}	48.38 {3.218, 93.54}	107.1 {103, 111.2}
Layered PETG/TPU-A *	751.4 {556.4, 946.4}	864.3 {846.1, 882.5}	−33.72 {−61.38, −6.061}	84.13 {81.55, 86.71}

* Assuming the average value for the following quantities: *ρ* = 1235 kg/m^3^, *υ* = 0.45.

**Table 3:** Poisson’s ratio results for tested materials.

Material	Poisson’s Ratio (*ν*)
PLA	0.39
PETG	0.42
TPU-A	0.49
TPU-C	0.49

**Table 4:** Vibration testing and FE results for single-material cantilever plates. Values are given in the following format: Average ± Standard Deviation.

Material	Method	Mode	*i* = 1	*i* = 2	*i* = 3
PETG	Experimental	$f_{i}$ (Hz)	16.22 ± 0.64	78.43 ± 1.91	99.78 ± 0.45
		$\zeta_{i}\;$ (%)	1.16 ± 0.54	1.17 ± 0.16	1.26 ± 0.13
	Fixed Modulus *	$f_{i}$ (Hz)	16.01 ± 0.10	64.64 ± 0.41	99.60 ± 0.62
		$\zeta_{i}\;$ (%)	1.68 ± 0.20	1.68 ± 0.20	1.68 ± 0.20
	Frequency-Dependent Modulus **	$f_{i}$ (Hz)	16.08 ± 0.07	68.16 ± 0.75	109.05 ± 1.73
		$\zeta_{i}\;$ (%)	1.69 ± 0.14	1.82 ± 0.33	1.90 ± 0.46
TPU-A	Experimental	$f_{i}$ (Hz)	15.44 ± 0.03	64.09 ± 0.12	102.9 ± 0.38
		$\zeta_{i}\;$ (%)	6.63 ± 0.13	5.86 ± 0.10	6.02 ± 0.47
	Fixed Modulus *	$f_{i}$ (Hz)	13.25 ± 1.34	51.66 ± 5.21	82.02 ± 8.27
		$\zeta_{i}\;$ (%)	7.76 ± 0.32	7.76 ± 0.32	7.76 ± 0.32
	Frequency-Dependent Modulus **	$f_{i}$ (Hz)	13.39 ± 0.67	66.32 ± 6.97	126.37 ± 17.9
		$\zeta_{i}\;$ (%)	7.64 ± 0.21	5.45 ± 0.08	4.35 ± 0.12

* Using mesh of 20 × 20 elements (1323 DOF). ** Using mesh of 15 × 15 elements (768 DOF).

**Table 5:** Vibration testing and FE results for graded cantilever plates. Values are given in the following format: Average ± Standard Deviation.

Material	Method	Mode	*i* = 1	*i* = 2	*i* = 3
PETG/TPU-A 1y	Experimental	$f_{i}$ (Hz)	17.97 ± 0.39	69.70 ± 1.72	105.46 ± 1.75
		$\zeta_{i}\;$(%)	1.71 ± 0.16	3.24 ± 0.89	6.93 ± 0.89
	Fixed Modulus *	$f_{i}$ (Hz)	15.69 ± 0.35	58.54 ± 3.04	88.56 ± 5.77
		$\zeta_{i}\;$(%)	2.74 ± 0.03	4.65 ± 0.17	5.48 ± 0.16
	Frequency-Dependent Modulus **	$f_{i}$ (Hz)	15.81 ± 0.19	67.68 ± 3.27	118.40 ± 9.39
		$\zeta_{i}\;$(%)	2.66 ± 0.03	3.37 ± 0.15	3.31 ± 0.27
PETG/TPU-A 2y	Experimental	$f_{i}$ (Hz)	16.33 ± 0.54	67.92 ± 1.65	105.53 ± 1.29
		$\zeta_{i}\;$(%)	3.72 ± 0.91	3.21 ± 0.15	4.06 ± 0.64
	Fixed Modulus *	$f_{i}$ (Hz)	14.77 ± 0.74	58.11 ± 2.91	94.42 ± 3.32
		$\zeta_{i}\;$(%)	4.58 ± 0.22	4.56 ± 0.04	3.62 ± 0.06
	Frequency-Dependent Modulus **	$f_{i}$ (Hz)	14.95 ± 0.37	67.34 ± 3.56	113.47 ± 4.82
		$\zeta_{i}\;$(%)	4.43 ± 0.09	3.50 ± 0.23	2.43 ± 0.40
TPU-A/PETG 1y	Experimental	$f_{i}$ (Hz)	14.10 ± 0.25	64.48 ± 0.66	100.45 ± 0.90
		$\zeta_{i}\;$(%)	5.27 ± 0.12	4.60 ± 0.90	3.22 ± 1.16
	Fixed Modulus *	$f_{i}$ (Hz)	13.45 ± 1.22	56.55 ± 3.58	92.57 ± 4.38
		$\zeta_{i}\;$(%)	7.13 ± 0.33	5.38 ± 0.22	4.36 ± 0.18
	Frequency-Dependent Modulus **	$f_{i}$ (Hz)	13.61 ± 0.61	66.91 ± 3.99	115.91 ± 6.45
		$\zeta_{i}\;$(%)	7.00 ± 0.20	3.82 ± 0.10	2.84 ± 0.38
TPU-A/PETG 2y	Experimental	$f_{i}$ (Hz)	15.75 ± 0.34	67.34 ± 1.31	101.56 ± 0.67
		$\zeta_{i}\;$(%)	5.47 ± 0.95	3.42 ± 0.14	4.35 ± 0.46
	Fixed Modulus *	$f_{i}$ (Hz)	14.24 ± 0.94	58.22 ± 2.86	86.83 ± 6.58
		$\zeta_{i}\;$(%)	5.58 ± 0.29	4.50 ± 0.06	6.21 ± 0.26
	Frequency-Dependent Modulus **	$f_{i}$ (Hz)	14.42 ± 0.47	67.38 ± 3.51	120.91 ± 11.58
		$\zeta_{i}\;$(%)	5.42 ± 0.15	3.46 ± 0.23	3.48 ± 0.24

* Using 20 × 21 elements (1386 DOF). ** Using 15 × 15 elements (816 DOF) for 1y, and 15 × 14 elements (720 DOF) for 2y.

## Data Availability

The original contributions presented in this study are included in the article. Further inquiries can be directed to the corresponding author.
